# Rosuvastatin Improves Vaspin Serum Levels in Obese Patients with Acute Coronary Syndrome

**DOI:** 10.3390/diseases6010009

**Published:** 2018-01-16

**Authors:** Hayder M. Al-kuraishy, Ali I. Al-Gareeb, Ali K. Al-Buhadilly

**Affiliations:** Department of Pharmacology, Toxicology, and Medicine, College of Medicine Al-Mustansiriyah University, P.O. Box 14132 Baghdad, Iraq; dr.alialgareeb78@yahoo.com (A.I.A.-G.); alikadm1977@yahoo.com (A.K.A.-B.)

**Keywords:** vaspin, acute coronary syndrome, NSTEMI, STEMI

## Abstract

Adipose tissue-derived serine protease inhibitor (vaspin), which has endocrine and local roles in atherosclerosis growth, is also synthesized by adipose tissue; it was found that vaspin was negatively correlated with blood pressure in obese patients, while vaspin levels were decreased in endothelial dysfunction. The aim of the present study was to determine rosuvastatin modulation effects on serum vaspin levels in acute coronary syndrome (ACS) with class I obesity. A total number of seventy patients with acute coronary syndrome previously and currently treated with rosuvastatin was compared to 40 patients with IHD not treated by rosuvastatin as a control. Vaspin serum levels were higher in rosuvastatin-treated patients with acute coronary syndrome compared to the patients with acute coronary syndrome not treated by rosuvastatin, *p* < 0.01. Additionally, in the rosuvastatin-treated group, patients with STEMI showed higher vaspin serum levels compared to NSTEMI *p* < 0.01. Conclusion: Rosuvastatin significantly increases vaspin serum levels in acute coronary syndrome.

## 1. Introduction 

Acute coronary syndrome (ACS) is a cluster of pathological conditions due to a reduction in coronary blood flow caused by coronary thrombosis and/or atherosclerosis, leading to myocardial ischemia and necrosis [1]. Acute coronary syndrome includes ST-elevation myocardial infarction (STEMI) 30%, non-ST elevation myocardial infarction (NSTEMI) 25%, and unstable angina 38%; these are classified according to electrocardiographic changes in ST-segment [2]. Acute coronary syndrome should be differentiated from stable angina (crescendo angina); in addition, new onset angina should be regarded as part of ACS [3]. Acute myocardial infarction (MI) is known as myocardial cell death (necrosis) because of prolonged myocardial ischemia, STEMI occurs when the coronary artery thrombus is initiated rapidly at coronary vascular wall injury, which can be triggered by many factors, including hypertension, cigarette smoking and dyslipidemia [4]. Unstable angina/non-ST-elevation MI patients usually have numerous vulnerable plaques at risk of disruption and rupture, so platelet aggregation in acute coronary syndromes (ACS) leads to unstable plaque [5]. The damage to the full thickness of the heart muscle is an indicator of increased damage percentage, while the partial thickness of the heart muscle damage is called NSTEMI [6].

ACS is associated with inflammatory and non-inflammatory risk factors; high blood levels of C-reactive protein (CRP) may be linked with risk of coronary artery disease and having a heart attack [7]. In healthy people without hyperlipidemia but with increased CRP levels, the statin drugs rosuvastatin significantly reduced the acute cardiovascular events [8,9]. 

Visceral Adipose Tissue-Derived Serpin (vaspin) is a novel adipokine that is expressed mainly in visceral white adipose tissue [10]. The up-regulation of vaspin synthesis may signify a response to the antagonizing action of fat-derived proteases that antagonize the insulin action; therefore, up-regulation of vaspin expression may be regarded as a defense mechanism against insulin resistance [11]. Additionally, vaspin was negatively correlated with blood pressure in obese patients, while vaspin levels were decreased in patients with impaired endothelial function [12]. Inflammation was thought to be one of the major causes of early atherosclerosis and its complications [13]. Recently, a study predicted that adipokines including vaspin have a local role in preventing the progression of atherosclerotic growth of [14]. 

Class I obesity is defined by the WHO as moderate obesity where body mass index (BMI) range is 30–35 kg/m^2^; obesity is positively correlated with the incidence of the acute coronary syndrome and vaspin serum levels [15].

Statins are the most broadly used lipid-lowering agents in patients with dyslipidemia, they reduce the cardio-metabolic risk factors, morbidity and mortality of cardiovascular diseases independent of their lipid-lowering effect. These effects were observed along with an augmentation in vaspin serum levels [16].

Rosuvastatin is a HMG-CoA reductase inhibitor indicated for dyslipidemia and approved in 2010 by the FDA for primary prevention of ACS due to a reduction in the cardiovascular risk factors regardless of lipid profile levels [17].

Therefore, the aim of the present study was to determine the effect of rosuvastatin on vaspin serum levels in obese patients with acute coronary syndrome.

## 2. Patient and Methods

In this cohort study, a total number of seventy patients (50 males, 20 females) previously and currently treated with rosuvastatin—25 patients with unstable angina pectoris (12 male and 13 female), 25 patients with STEMI (22 male and 3 female) and 20 patients with NSTEMI (16 male and 4 female)—were enrolled in the study and compared to 40 patients with IHD not treated by rosuvastatin, as a control. Each patient was clinically examined by the consultant, and the diagnosis was achieved by electrocardiograph ECG, cardiac enzymes, and cardiac Troponin (cTnI). The inclusion criteria were: unstable angina pectoris, STEMI and NSTEMI patients with recent acute myocardial infarction admitted to the coronary care unit (CCU). The exclusion criteria were patients with valvular heart diseases, malignant diseases, acute infection, inflammatory disorders, blood disorders, advanced renal disease, liver disease, smoking and diabetes mellitus. All enrolled patients and controlled subjects gave written informed approval before their participation. The procedures were prepared according to the Declaration of Helsinki. This study was approved by the Clinical Research, Ethical Committee, College of Medicine, Al-Mustansiriyiah University, Baghdad-Iraq.

## 3. Sample Collections and Anthropometric Profiles

After the interview, medical history, current drug pharmacotherapy and anthropometric measures were examined. Body mass index was estimated as kg/m^2^, waist and hip circumferences were recorded, and the waist-hip ratio was calculated. Waist-hip ratio was determined by dividing the waist (cm) by the hip (cm), using a cutoff level <0.85 in females and <0.9 in males [18]. Ten milliliters of venous blood was withdrawn at 9 a.m. after overnight fasting, into a plain tube 5 mL (for routine investigations) and into an EDTA tube 5 mL for vaspin and (cTnI) estimations.

Vaspin and cTnI serum levels were determined by ELISA KIT method (vaspin inhibitor) in pg/mL at 450 nm and human troponin-I (TNNI2) in pg/mL at 450 nm, respectively.

Assessment of lipid profile: triglyceride (TG), total cholesterol (TC) and high-density lipoprotein (HDL) were assessed by specific ELISA kits; from this profile, we can measure the following: [19]

Atherogenic index (AI) = log (TG/HDL), when TG and HDL measured in mmol/L.

LDL = (TC)-(HDL)-(TG)/5.

VLDL = TG/5.

Cardiac risk ratio (CRR) = TC/HDL.

## 4. Statistical Analysis

Data analysis was done using SPSS (IBM SPSS, Stastics for Window, Version 22.00; 2014, Armonk, NY, USA: IBM Corp.). Results are expressed as mean ± SD, number and percentage. Unpaired student *t* test was used for estimation of differences between two different groups and one way ANOVA test for estimation the differences among treated groups in terms of 95% confidence interval and *t* value. Pearson correlation coefficient was used to evaluate the correlation of vaspin serum levels with other study parameters. The results were regarded as significant when *p* < 0.05.

## 5. Results

Baseline characteristics of the present study between the study group (the rosuvastatin-treated group) and the control group (the rosuvastatin-free group) demonstrated non-significant differences in most of the patient variables *p* > 0.05 but, there were significant differences in the presentation of hypertension and current statin therapy *p* < 0.0001, in addition to minor differences regarding other pharmacotherapy *p* < 0.05, Table 1.

Vaspin serum levels were higher in the study group compared to the control group *p* < 0.01, whereas there were non-significant differences in serum cardiac troponin-I serum level, diastolic blood pressure and blood glucose *p* > 0.05. Moreover, the rosuvastatin-treated group revealed significant differences among other biochemical variables compared to the control group (the rosuvastatin-free group), particularly on high-sensitivity CRP, Table 2.

Regarding the intra-group and inter-group differences, unstable angina versus control showed a significant difference in vaspin and cardiac troponin-I sera levels in addition to the other cardio-metabolic variables, except in cardiac risk ratio (CCR); in the same manner, these differences were found between STEMI and NSTEMI versus control. In the rosuvastatin-treated group, patients with STEMI showed higher vaspin serum levels and lower atherogenic index compared to NSTEMI Table 3.

Therefore, vaspin serum levels were higher in rosuvastatin-treated patients with acute coronary syndrome compared to the patients with acute coronary syndrome not treated with rosuvastatin. Additionally, in the rosuvastatin-treated group, patients with STEMI showed higher vaspin serum levels compared to NSTEMI.

**Figure 1:**
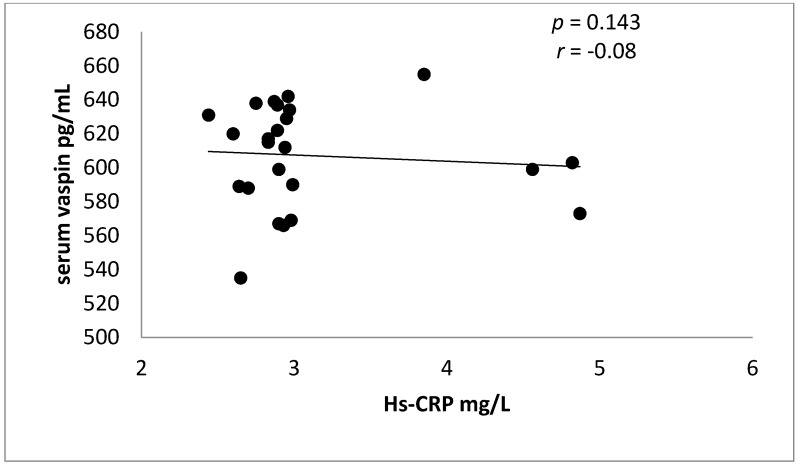
Negative correlation between vaspin serum levels and Hs-CRP in rosuvastatin-treated ACS.

Indeed, there is a non-significant negative correlation between vaspin serum levels and Hs-CRP in ACS in rosuvastatin-treated patients Figure 1.

**Figure 2:**
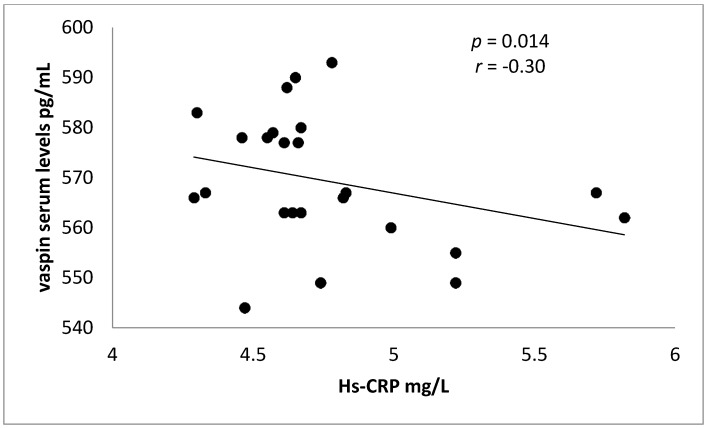
Negative correlation between vaspin serum levels and Hs-CRP in rosuvastatin-free ACS patients.

Meanwhile, in control patients (rosuvastatin free), there was a significant negative correlation between vaspin serum levels and Hs-CRP in ACS, see Figure 2.

Vaspin serum levels were positively correlated with cTn-I (*p* < 0.001) and HDL (*p* = 0.0003), but negatively correlated with total cholesterol (*p* = 0.007), triglyceride (*p* = 0.04) and atherogenic index (*p* = 0.04) in patients with STEMI. Meanwhile, in patients with NSTEMI, vaspin serum levels were mainly correlated with cTn-I, total cholesterol and HDL *p* < 0.01; while in patients with unstable angina, vaspin serum levels were only correlated with total cholesterol and HDL *p* < 0.01. In the control patients (rosuvastatin-free patients), vaspin serum levels were correlated with cardio-metabolic risk profile *p* < 0.05, see Table 4.

**Figure 3:**
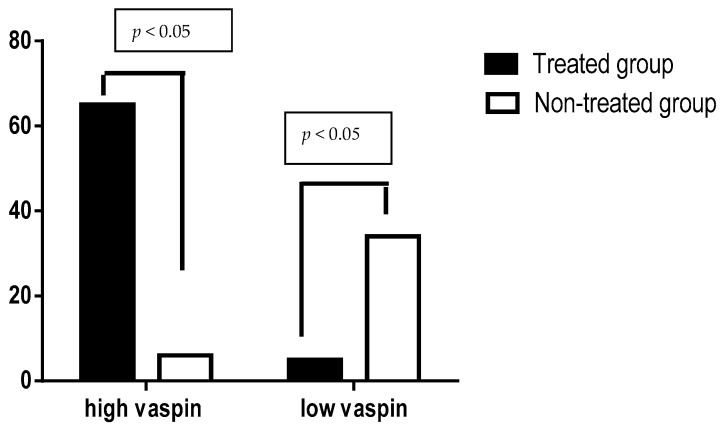
Sensitivity and specificity of vaspin serum levels in patients with acute coronary syndrome treated with or without rosuvastatin.

Regarding the sensitivity and specificity of vaspin serum levels in patients with ACS, high vaspin serum levels( more than 550 pg/mL) were found in 65 patients (treated group) compared to 6 patients (control group) *p* < 0.05 (positive predictive value 0.915 with 95% CI 0.818−0.965), whereas low vaspin serum levels (less than 550 pg/mL) were found in 5 patients (treated group) compared to 34 patients (control group) *p* < 0.05 (negative predictive value 0.871 with 95% CI 0.717−0.951), see Figure 3. 

**Figure 4:**
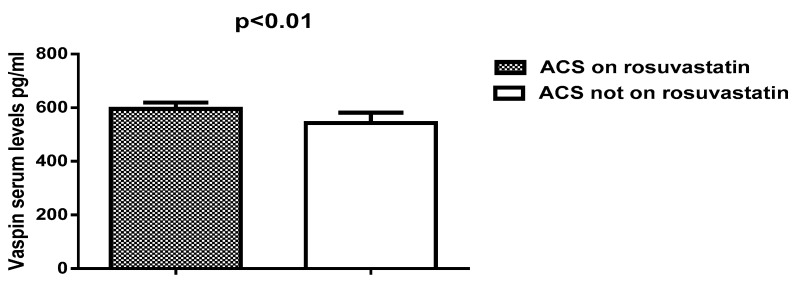
Vaspin serum levels in patients with ACS regarding rosuvastatin therapy.

Thus, vaspin serum levels were high in patients with ACS on rosuvastatin therapy (595.62 ± 23.65) compared to patients with ACS not on rosuvastatin therapy (542.75 ± 38.95), see Figure 4.

## 6. Discussion

Vaspin was initially recognized as an adipokine, mainly secreted from visceral adipose tissue in Otsuka Long-Evans Tokushima Fatty (OLETF), which is an animal model of obesity and diabetes mellitus [20]. Higher vaspin mRNA expression is correlated with type 2 diabetes mellitus, obesity and insulin resistance; this increment in vaspin serum levels leads to improvements in glucose metabolism and insulin sensitivity in visceral adipose tissue, but the main molecular mechanism of vaspin is unidentified [21]. Moreover, vaspin plays an important role in the modulation of coronary vessel homeostasis, since vaspin has a local effect on coronary vascular endothelium [22].

The present study demonstrated higher vaspin serum levels in both the study and control groups; given that the patients in both groups were obese class I type (BMI > 30 kg/m^2^), this corresponds to the study by Cho et al., which revealed a significant correlation between vaspin levels and BMI, since vaspin concentrations were increased and correlated with elevated total body fat percentage. For this reason, vaspin concentrations can be regarded as an indicator of obesity [23]. The waist-hip ratio of enrolled patients in the present study was high in both enrolled men and women. This ratio was associated with a high vaspin concentration; this finding correlates with that of the Amouzad et al., 2014 study, which revealed a positive link between elevated waist-hip ratio and serum vaspin concentrations in obesity and metabolic syndromes [24]. Contradictory studies have reported low vaspin serum concentration in obesity compared to normal healthy volunteers [25], which does not correspond with the findings of the present study.

Serum vaspin concentrations of our work were relatively low in patients with ACS, which is consistent with the study by Kobat et al., which demonstrated low vaspin serum levels in patients with ACS and coronary atherosclerosis [26].

The current study also revealed a higher vaspin serum concentration in patients with ACS previously and currently treated with rosuvastatin compared with patients with ACS not previously and currently treated by statins; this result is supported by the 2011 study by Kadoglou et al., which revealed that the pleiotropic effects of statin may increase vaspin serum levels in patients with ischemic heart disease [27].

Moreover, serum vaspin concentrations may be equal in unstable angina and NSTEMI, but there was a significant difference in serum vaspin concentrations between NSTEMI and STEMI. When it is higher in STEMI, this may be due to rosuvastatin effects, and all of the enrolled patients were obese since vaspin serum levels are higher in obesity and are provoked by statins therapy, because statins produced more significant anti-inflammatory effects in STEMI than NSTEMI [28].

Rosuvastatin has potent immuno-modulatory and anti-inflammatory effects due to its inhibition of pro-inflammatory mediators, including tumor necrosis factor (TNF-α), interleukin-6 and C-reactive protein [29], which corresponds with our findings, which revealed a decline in highly sensitive C-reactive protein (hs-CRP) in rosuvastatin-treated patients compared to non-treated patients with ACS. This may explain the decremental effects of rosuvastatin on morbidity of cardio-metabolic risk profile independent of/from lipid-lowering property, which is called the pleiotropic effect [30].

Consequently, the 2011 study of Kodaglou et al. pointed out that inflammatory pathways are the link between obesity and coronary heart diseases, vaspin serum levels showed an inverse association with acute cardiac ischemic events, so low vaspin concentrations were correlated with ACS severity, which is suggestive of the cardio-protective effects of vaspin [31].

Vaspin plays an important role in the prevention of vascular and coronary endothelial injuries and inflammation through down-regulation of intracellular adhesion molecules induced by TNF-α, inhibition of reactive oxygen species, inhibition of platelet-derived growth factor, and suppression of free radical-dependent p38/HSP27 activation [32,33]. Thus, rosuvastatin, like other statins, leads to direct or indirect anti-inflammatory effect via increment in vaspin serum levels that ameliorates acute inflammatory changes in ACS [34], as presented in the present study through elevation of vaspin serum levels and decrement in Hs-CRP. 

Additionally, vaspin shows a diurnal variation, high at morning and fasting, and low at the postprandial period; additionally, high vaspin serum levels were reported to be higher in the Asian population [35], these variations may explain high vaspin serum levels in the current study, because all of the selected patients were Asians, and blood sampling was done in the morning to exclude these variations.

Indeed, hs-CRP serum level is an inflammatory marker that increased within two days following ACS, but not in unstable angina; this increment continued for three months subsequent to myocardial infarction [36]. Rosuvastatin significantly reduces Hs-CRP serum levels through its anti-inflammatory properties, as shown in the present study.

Finally, the present study illustrated the inverse correlation of vaspin serum levels with most cardio-metabolic risk profiles in patients with acute coronary syndromes not treated with rosuvastatin, compared to rosuvastatin-treated patients. These findings are in agreement with many studies showing that vaspin levels were found to have an inverse link with the cardio-metabolic events, signifying the protective effect of vaspin in the prevention of coronary atherosclerosis and amelioration of cardiac risk factors [37,38]. Unfortunately, this study did not measure insulin levels; additionally, gender and race differences were not evaluated. These limitations may be a project for future research.

## 7. Conclusions

Rosuvastatin significantly increases vaspin serum levels in acute coronary syndrome.

## Figures and Tables

**Table 1:** Baseline characteristics of the present study.

Variables	Rosuvastatin Group	Control Group	*p* Value
	(*n* = 70)	(*n* = 40)	
**Age**	47.87 ± 12.72	49.85 ± 11.83	0.52
**Gender**			
**M:F ratio**	(71.42:28.57)%	(22:18) %	-
**BMI**	31.44 ± 7.81	32.39 ± 6.77	0.59
**W-H ratio**			
**Men**	1.32 ± 0.67	1.39 ± 0.77	0.71
**Women**	0.88 ± 0.31	0.91 ± 0.11	0.67
**Hypertension**	70 (100%)	20 (100%)	<0.0001 **
**ACS**			
**STEMI**	25 (35.71%)	8 (40%)	0.72
**NSTEMI**	25 (35.71%)	10 (50%)	0.24
**UA**	20 (28.57%)	2 (10%)	0.08
**Troponin positive**	67 (95.71%)	18 (90%)	0.32
**Troponin negative**	3 (4.28%)	2 (10%)	0.32
**Dyslipidemia**	64 (44.8%)	18 (90%)	0.84
**Duration of IHD (years)**	8.42 ± 2.28	7.44 ± 2.39	0.11
**Diabetes mellitus**	3 (4.28%)	2 (10%)	0.32
**Pharmacotherapy**			
**Anticoagulant**	44 (62.58%)	17 (85%)	0.06
**Antiplatelet**	70 (100%)	18 (90%)	0.0072
**ACEIs**	44 (62.58%)	18 (90%)	0.02 *
**Statins**	70 (100%)	-	<0.0001 **
**CCB**	22 (31.42%)	12 (60%)	0.02 *
**β-blockers**	6 (8.57%)	5 (25%)	0.04 *
**Insulin**	3 (4.28%)	2 (10%)	0.32
**Complications**	10 (15.71%)	4 (20%)	0.53
**Shock**	2 (2.85%)	1 (5%)	0.63
**Heart failure**	5 (3.5%)	2 (10%)	0.67
**Cardiac aneurysm**	1 (1.42%)	1 (5%)	0.33
**Death**	2 (2.85%)	1 (5%)	0.33

Data presented as mean ± SD, number and %, * *p* < 0.05, ** *p* < 0.01 versus control. W-H ratio: waist-hip ratio.

**Table 2:** Comparison between study and control groups in vaspin serum levels and other cardio-metabolic risk variables.

Cardio-Metabolic Variables	Study Group (*n* = 70)	Control Group (*n* = 40)	*t* Value	95% CI Upper-Lower Limits	*p* Value
**Serum cTn-I pg/mL)**	74.54 ± 13.32	77.64 ± 12.22	−0.98	3.33–9.53	0.33
**Serum vaspin(pg/mL)**	603.83 ± 18.13	542.75 ± 38.95	6.8	79.72–42.33	<0.0001 **
**TC (mg/dL)**	199.28 ± 20.49	266.43 ± 16.59	−15.1	−134.29	<0.0001 **
**TG (mg/dL)**	166.83 ± 17.34	254.73 ± 22.82	−15.95	−175.78	<0.0001 **
**LDL (mg/dL)**	118.80 ± 8.75	164.71 ± 13.59	−14.28	−81.81	<0.0001 **
**HDL(mg/dL)**	47.11 ± 9.86	50.76 ± 7.34	−1.8	0.43–7.73	0.078
**VLDL (mg/dL)**	33.36 ± 5.83	50.94 ± 6.42	−11.01	−35.15	<0.0001 **
**AI**	0.189 ± 0.012	0.341 ± 0.021	−30.95	−0.3	<0.0001 **
**CRR**	4.23 ± 1.44	5.24 ± 1.98	−2.12	−2.01	0.04 *
**SBP (mmHg)**	166.54 ± 21.54	155.87 ± 19.63	2.09	21.02–0.31	0.043 *
**DBP (mmHg)**	92.23 ± 22.69	87.67 ± 13.75	1.11	12.78–3.66	0.27
**FBG (mg/dL)**	101.65 ± 11.93	97.87 ± 8.97	1.53	8.75–1.19	0.132
**PPG(mg/dL)**	128.64 ± 11.83	132.64 ± 12.74	−1.25	2.50–10.50	0.21
**Hs-CRP (mg/L)**	2.95 ± 0.45	4.65 ± 1.84	−4.09	−3.39	0.0006 **

Data presented as mean ± SD, * *p* < 0.05, ** *p* < 0.01 versus control; cTn-I: cardiac troponin-I; TG: triglyceride; TC: total cholesterol; LDL: low density lipoprotein; HDL: high density lipoprotein; VLDL: very low density lipoprotein; AI: atherogenic index; CRR: cardiac risk ratio; SBP: systolic blood pressure; DBP: diastolic blood pressure; FBG: fasting blood glucose; PPG: postprandial glucose; Hs-CRP: highly sensitive CRP.

**Table 3:** Intra and inter-groups difference in cardio-metabolic variables.

Cardio-Metabolic Variables	STEMI (*n* = 25)	NSTEMI (*n* = 25)	Unstable Angina (*n* = 20)	Control (*n* = 40)
**Serum cTn-I (pg/mL)**	74.12 ± 12.45	72.65 ± 22.49	54.45 ± 23.53 **	77.64 ± 12.22
**Serum vaspin (pg/mL)**	611.32 ± 33.64 **	588.67 ± 22.29 **	586.87 ± 33.72 **	542.75 ± 38.95
**TC (mg/dL)**	197.28 ± 21.77 **	192.99 ± 19.64 **	193.64 ± 20.65 **	266.43 ± 16.59
**TG (mg/dL)**	160.83 ± 19.42 **	167.54 ± 18.74 **	166.83 ± 16.84 **	254.73 ± 22.82
**LDL (mg/dL)**	117.78 ± 8.75 **	113.85 ± 9.98 **	112.63 ± 13.83 **	164.71 ± 13.59
**HDL (mg/dL)**	47.33 ± 9.77	45.63 ± 8.99	45.55 ± 8.33 *	31.76 ± 7.34
**VLDL (mg/dL)**	32.16 ± 5.88 **	33.50 ± 4.34 **	33.57 ± 4.22 **	50.94 ± 6.42
**AI**	0.171 ± 0.011 **	0.205 ± 0.014 **	0.204 ± 0.012 **	0.341 ± 0.021
**CRR**	4.16 ± 1.65	4.22 ± 1.99	4.25 ± 1.86	5.24 ± 1.98
**SBP (mmHg)**	162.66 ± 20.33	165.62 ± 19.82	164.76 ± 18.93	155.87 ± 19.63
**DBP (mmHg)**	91.20 ± 20.39	93.53 ± 20.37	93.77 ± 20.53	87.67 ± 13.75
**Pulse pressure (mmHg)**	71.46 ± 11.76	72.09 ± 10.74	70.99 ± 11.55	68.20 ± 9.33
**FBG (mg/dL)**	101.44 ± 10.98	100.54 ± 10.71	104.61 ± 9.88	97.87 ± 8.97
**PPG (mg/dL)**	129.60 ± 10.82	127.55 ± 9.76	126.83 ± 9.38	132.64 ± 12.74
**Hs-CRP (mg/L)**	2.93 ± 0.44 **	2.89 ± 0.45 **	1.44 ± 0.11 **	4.65 ± 1.84

Data presented as mean ± SD, * *p* < 0.05, ** *p* < 0.01 versus control, *p* < 0.01 (STEMI versus NSTEMI); cTn-I: cardiac troponin-I; TG: triglyceride; TC: total cholesterol; LDL: low density lipoprotein; HDL: high density lipoprotein; VLDL: very low density lipoprotein; AI: atherogenic index; CRR: cardiac risk ratio; SBP: systolic blood pressure; DBP: diastolic blood pressure; FBG: fasting blood glucose; PPG: postprandial glucose; Hs-CRP: highly sensitive CRP.

**Table 4:** Pearson correlation for vaspin serum levels with the cardio-metabolic risk factors in patients with acute coronary syndrome.

Variables	STEMI (*n* = 25)		NSTEMI (*n* = 25)		UA (*n* = 20)		Control (*n* = 40)	
	*r*	*p*	*r*	*p*	*r*	*p*	*r*	*p*
**Serum cTn-I (pg/mL)**	0.87	0.001 *	0.77	0.0001 *	0.44	NS	0.94	0.0001 *
**TC (mg/dL)**	−0.52	0.007 *	−0.67	0.0002 *	−0.67	0.001 *	−0.56	0.007 *
**TG (mg/dL)**	−0.41	0.04 !	−0.32	NS	−0.36	NS	−0.75	0.001 *
**LDL (mg/dL)**	−0.33	NS	−0.32	NS	0.38	NS	−0.63	0.0002 *
**HDL (mg/dL)**	0.66	0.0003 *	0.69	0.0002 *	0.71	0.0004 *	0.51	0.008 *
**VLDL (mg/dL)**	−0.31	NS	−0.34	NS	−0.38	NS	−0.42	0.006 *
**AI**	−0.41	0.04 !	−0.35	NS	−0.41	NS	−0.51	0.0007 *
**CRR**	−0.34	NS	−0.31	NS	−0.37	NS	−0.41	0.008 *
**SBP (mmHg)**	−0.33	NS	−0.36	NS	−0.32	NS	−0.42	0.006 *
**DBP (mmHg)**	−0.37	NS	−0.30	NS	−0.22	NS	−0.41	0.008 *
**FBG (mg/dL)**	−0.28	NS	−0.32	NS	−0.30	NS	−0.33	0.03 !

Pearson correlation (*r*); * *p* < 0.01; ! *p* < 0.05; NS: non-significant; UA: unstable angina; cTn-I: cardiac troponin-I; TG: triglyceride; TC: total cholesterol; LDL: low density lipoprotein; HDL: high density lipoprotein; VLDL: very low density lipoprotein; AI: atherogenic index; CRR: cardiac risk ratio; SBP: systolic blood pressure; DBP: diastolic blood pressure; FBG: fasting blood glucose.
